# Genetic architecture of pollination syndrome transition between hummingbird-specialist and generalist species in the genus *Rhytidophyllum* (Gesneriaceae)

**DOI:** 10.7717/peerj.1028

**Published:** 2015-06-18

**Authors:** Hermine Alexandre, Justine Vrignaud, Brigitte Mangin, Simon Joly

**Affiliations:** 1 Institut de Recherche en Biologie Végétale, Université de Montréal, Montreal, QC, Canada; 2 INRA, Laboratoire des Interactions Plantes-Microorganismes (LIPM) UMR, Castanet-Tolosan, France; 3 CNRS, Laboratoire des Interactions Plantes-Microorganismes (LIPM) UMR, Castanet-Tolosan, France; 4 Montreal Botanical Garden, Montreal, QC, Canada

**Keywords:** Pollination syndrome, Geometric morphometrics, QTL, Genotyping by sequencing, Plant mating systems, Floral evolution

## Abstract

Adaptation to pollinators is a key factor of diversification in angiosperms. The Caribbean sister genera *Rhytidophyllum* and *Gesneria* present an important diversification of floral characters. Most of their species can be divided in two major pollination syndromes. Large-open flowers with pale colours and great amount of nectar represent the generalist syndrome, while the hummingbird-specialist syndrome corresponds to red tubular flowers with a less important nectar volume. Repeated convergent evolution toward the generalist syndrome in this group suggests that such transitions rely on few genes of moderate to large effect. To test this hypothesis, we built a linkage map and performed a QTL detection for divergent pollination syndrome traits by crossing one specimen of the generalist species *Rhytidophyllum auriculatum* with one specimen of the hummingbird pollinated *R. rupincola*. Using geometric morphometrics and univariate traits measurements, we found that floral shape among the second-generation hybrids is correlated with morphological variation observed between generalist and hummingbird-specialist species at the genus level. The QTL analysis showed that colour and nectar volume variation between syndromes involve each one major QTL while floral shape has a more complex genetic basis and rely on few genes of moderate effect. Finally, we did not detect any genetic linkage between the QTLs underlying those traits. This genetic independence of traits could have facilitated evolution toward optimal syndromes.

## Introduction

The flower is a key innovation often invoked to explain the radiation and evolutionary success of angiosperms (Stebbins, 1970). Flowers present variable traits such as shape, colour, flowering time from which it is often possible to distinguish groups of traits that evolve jointly for the flower to be effectively pollinated by a given type of pollinator. These groups of traits are called pollination syndromes (Fenster et al., 2004). The selection for these syndromes is often so strong that it is possible to predict which type of pollinator a given plant species relies on via the observed syndrome. For instance, flowers can harbour very different traits depending on whether they are pollinated by wind or animals (Friedman & Barrett, 2009). In animal-pollinated species, major traits involved in pollination syndrome include corolla shape and colour, floral scent, as well as the amount and concentration of nectar produced, and variation in these traits enable species to be distinguish by different groups of pollinating animals. Rosas-Guerrero et al. (2014) reviewed floral traits of 417 species and showed that the concept of pollination syndrome can be very effective at predicting the pollinators of animal pollinated flowers, more so than for non-animal syndromes. Interestingly, syndrome predictability is more effective for tropical plants, probably because of lower pollinator population densities in the tropics that increase selection pressure (Rosas-Guerrero et al., 2014).

Pollination syndrome is a set of very dynamic and rapidly evolving characteristics, providing numerous examples of convergent evolution in many groups. In *Penstemon* (Plantaginaceae), for example, ornithophilous pollination evolved multiple times from insect pollinated flowers (Wilson et al., 2007). In *Ruellia*, insect pollination evolved repeatedly from the ancestral hummingbird pollination (Tripp & Manos, 2008). In *Gesneria* and *Rhytidophyllum* (Gesneriaceae), generalist and bat-pollinated species evolved several times from a hummingbird syndrome (Martén-Rodríguez et al., 2010). The tribe Sinningieae of the Gesneriaceae also shows an important lability of pollination modes, associated with evolution of traits such as corolla shape and colour (Perret et al., 2007). Because such transitions between syndromes are often linked with species diversification (reviewed in Van der Niet & Johnson, 2012), understanding how these transitions occur is critical for understanding angiosperms evolution.

Observations of such an important lability of flower characteristics, combined with the fact that flower diversification is often linked to species diversification, led us to wonder about the genetic basis of these traits. Studies of the genetic basis of phenotypic evolution are often focused on determining (i) if parallel phenotypic changes rely on parallel genomic evolution and (ii) if these major phenotypic transitions result from major changes at a limited number of genes or from minor changes of multiple genes (reviewed in Hendry, 2013). In addition, developmental constraints such as genetic interactions (epistasy) could be important to explain the convergence of different traits to form a particular syndrome. Similarly, there are potentially important roles for genetic correlations between traits and ecological factors—such as pollinator pressures—in the redundant evolution of floral phenotypes among different species. Indeed, the speed at which a population reaches its fitness optimum greatly depends on whether traits composing the pollination syndrome are genetically independent or linked. Three scenarios can be envisaged: (i) if traits are positively correlated, selection on one trait will affect variation at other traits in a positive way and the general fitness optimum should be reached rapidly; (ii) if traits are genetically independent, no developmental constraints should affect the evolution towards the optimum and the speed of adaptation will solely be influenced by the intensity of the selective pressure; and (iii) if traits are negatively correlated, selection at one trait will pull variation at other traits further from the fitness optimum, hence reducing the pace at which this optimum can be reached. Deciphering the degree of genetic correlation among traits is thus a first step toward understanding the relative role of selection versus intrinsic constraints in the evolution of phenotypes (Ashman & Majetic, 2006).

To answer these questions, a popular approach is to perform QTL detection on a hybrid population generated from parents with different pollination syndromes. Previous studies have shown that colour transition is generally explained by one major QTL (Quattrocchio et al., 1998; Yuan et al., 2013; Wessinger, Hileman & Rausher, 2014). In contrast, nectar volume and concentration frequently rely on numerous genomic regions each having a small to moderate effect on phenotype (Goodwillie, Ritland & Ritland, 2006; Galliot et al., 2006; Nakazato, Rieseberg & Wood, 2013). Flower shape variation was also shown to be generally caused by several QTLs with small to moderate effects, with frequent colocalization of those QTLs (reviewed in Hermann & Kuhlemeier, 2011). During the past several years, emerging next generation sequencing technologies have enabled the study of the genetic basis of adaptation in non-model species. Also, improvements of methods to study morphology, principally with geometric morphometrics, now enable to study the genetic basis and evolution of these complex characteristics (Klingenberg et al., 2001; Langlade et al., 2005; Klingenberg, 2010; Rogers et al., 2012; Franchini et al., 2014; Liu et al., 2014).

The closely related genera *Gesneria* and *Rhytidophyllum* consist of approximately 75 species and have rapidly diversified in the Antilles from a common ancestor that existed approximately 8 to 11 mya (Roalson, Skog & Zimmer, 2008). During this rapid species diversification, the group also simultaneously experienced a rapid diversification of floral traits. Floral shape, colour and nectar production have evolved jointly into three evolutionarily labile pollination syndromes (Martén-Rodríguez, Almarales-Castro & Fenster, 2009; Martén-Rodríguez et al., 2010): (i) species pollinated by hummingbirds that have red tubular flowers with diurnal nectar production, (ii) species pollinated by bats harbouring large pale flowers with a bell shape corolla and exhibit nocturnal nectar production, and (iii) generalist species that can either be pollinated by hummingbirds, bats or moths, have generally pale flowers (although often with various spots) with large openings but with a constriction in the corolla, and can have nocturnal and diurnal nectar production. It has been inferred that the hummingbird syndrome is the ancestral pollination mode whereas the bat and generalist syndromes evolved independently several times (with reversals back to the ancestral hummingbird syndrome having been tentatively identified) (Martén-Rodríguez et al., 2010). We intend here to identify the genetic basis of the pollination syndrome transition between the generalist and hummingbird-specialist species in *Rhytidophyllum* using QTL detection in a second-generation hybrid population. *Rhytidophyllum auriculatum* is a typical generalist species from Hispaniola and Puerto Rico, and harbours opened yellow flowers producing large amount of nectar. The second species, *R. rupincola*, is a hummingbird specialist with red and tubular flowers that produces only small quantities of nectar. Its endemism to Cuba (Skog, 1976; Martén-Rodríguez et al., 2010; J Clark, pers. comm., 2013) eliminates all potential for natural hybridization with *R. auriculatum*. According to Martén-Rodríguez et al. (2010), *R. auriculatum* most likely belongs to a group of generalist that evolved from an ancestral hummingbird syndrome, whereas *R. rupincola* likely represents a reversion to the ancestral hummingbird syndrome; the two species being closely related but not sister species.

In this study, we obtained anonymous genetic markers via next generation sequencing (NGS) and built a linkage map from a second generation (F2) hybrid population between *R. rupincola* and *R. auriculatum*. We then used geometric morphometrics to study floral shape and test whether QTLs underlying floral trait evolution are few or numerous and whether they are linked or not.

## Material and Methods

### Study system

*Rhytidophyllum auriculatum* (female parent) was crossed with *R. rupincola* (male parent) from specimens from the living collection of the Montreal Botanical Garden (Canada) in 2010 to obtain first-generation (F1) hybrids. An F1 individual was self-fertilized in 2011 to give a second-generation (F2) population of 177 individuals. In parallel, both parents were self-pollinated and gave several viable individuals.

### Phenotypic measurements

**Figure 1:**
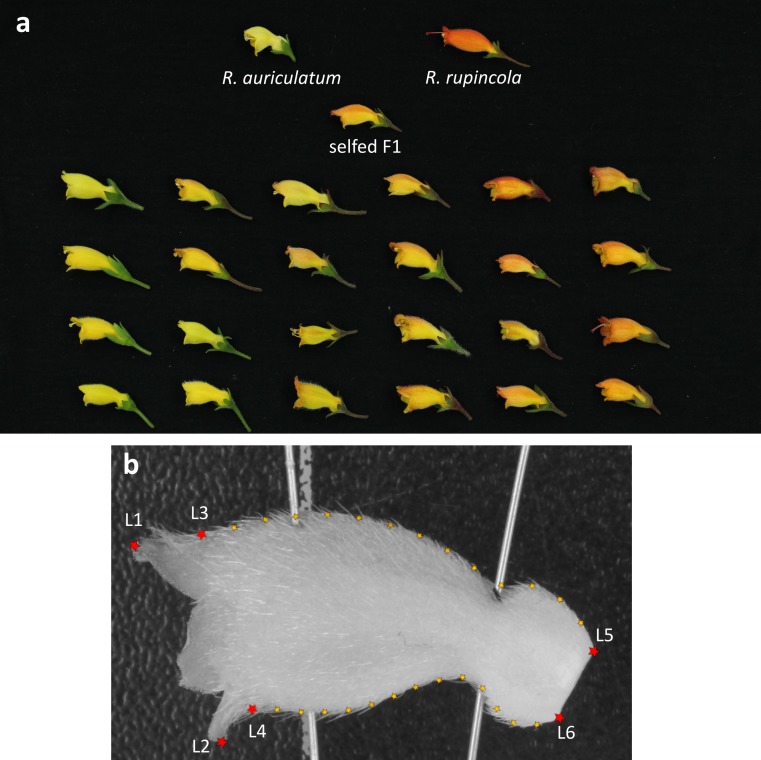
Measure of shape variation in the hybrid population and parents. (A) Flowers from both parents (top row), the self-pollinated F1 and samples from the F2 population; (B) position of landmarks on corolla pictures- red stars represent landmarks and small orange stars are semi-landmarks.

Phenotypic measures were performed from June of 2013 to April 2014 for morphological and colour traits because of a great heterogeneity of developmental rate in the population. Flower colour was treated as a binary trait: orange or yellow. Given the large variation in intensity and distribution of the orange colour on the corolla (Fig. 1), individuals were considered “orange” when some orange colour was observed on them. Corolla shape was analysed with geometric morphometrics methods designed to capture morphological characteristics of pollination syndromes without a priori hypotheses. In addition of allowing the determination of shape that is representative of a particular pollination mode, geometric morphometric methods have also been shown to be very efficient at revealing the genetic basis of complex morphological changes (Klingenberg et al., 2001).

**Figure 2:**
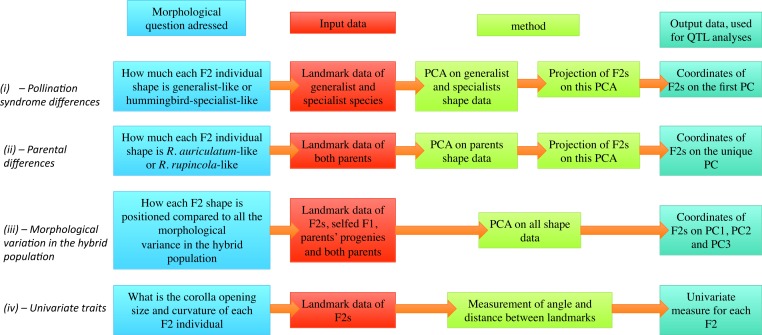
Diagram presentation of the four morphological measurement approaches.

For each individual, between one and three flowers were photographed. Each photo was analysed twice with the software TpsDIG2 (http://life.bio.sunysb.edu/morph/soft-dataacq.html), to evaluate variance due to manipulation errors in our analyses. Photographs from a different study (F Lambert, 2015, unpublished data) were also included to quantify shape variation in the whole *Gesneria/Rhytidophyllum* clade. This was done to characterize the aspects of shape that were the most significant to differentiate generalists from hummingbird specialists (see below). For these photographs, a single flower per individual was included. Six landmarks and 24 semi-landmarks were placed on each photo. Two landmarks were placed at the extremity of the petal lobes (L1, L2), two at the base of the petal lobes (L3, L4) and two at the base of the corolla (L5, L6). Semi-landmarks were evenly dispersed on the contour of the corolla between L3–L4 and L5–L6 (Fig. 1). Geometric morphometrics analyses were then performed in R (R Core Team, 2014) with packages shapes (Dryden, 2014), geomorph (Adams & Otárola-Castillo, 2013) and ade4 (Dray & Dufour, 2007). A general Procrustes superimposition of all the photos was performed with the function *gpagen* allowing for sliding semi-landmarks in the superimposition, and the mean coordinates of the landmarks and semi-landmarks per individual were extracted to get only one shape per individual. Morphology was then measured using four approaches to address the problem from different facets (see Fig. 2 for more details): (i—*Pollination syndrome differences*) A PCA (function *dudi.pca*) of nine generalist and nine hummingbird specialist species from the genera *Gesneria* and *Rhytidophyllum* (see Table S1 for details) was performed, and the F2 individuals were projected (function *suprow*) on the first PC that represents the shape difference between hummingbird-specialists and generalists. This approach estimates how much each F2 individual resembles hummingbird specialists or generalists. (ii—*Parental differences*) A PCA was performed on the two parents, giving only one principal component upon which the F2 individuals were projected. This approach measures how much each F2 individual resembles each parent. (iii—*Morphological variation in the hybrid population*) A PCA of the F2 population was performed (including the self-pollinated F1, both parents and three progenies of the self-pollinated parents), from which the scores of the F2 individuals were directly obtained. This approach allows investigating the genetic bases of the morphological variation observed in the F2 hybrid population. (iv—*Univariate traits*) Two univariate traits were extracted from the landmarks data before the Procrustes superimposition: the corolla tube opening corresponds to the distance between L3 and L4, and corolla curvature as the angle formed by the lines (L1–L2) and (L5–L6) (Fig. 1). Pictures from wild specimens were used to analyse shape only, without any size component because photos did not include a scale. Among the 141 individuals that gave flowers, four F2 individuals with abnormal flowers (disjoint petals or different flower shapes within an individual) and seven individuals presenting flowers with more or less than 5 petal lobes were discarded from the phenotypic measures, leaving 130 individuals for shape analysis.

Measurements of nectar volume were performed between November and December 2014. Nectar was sampled in early afternoon after flower opening, which generally occurs two days after flower opening. This time was chosen because nectar is released mainly at dawn and dusk in *Gesneria* and *Rhytidophyllum* (Martén-Rodríguez & Fenster, 2008), and because no nectar production was observed during the day for the parental species. To sample nectar, the flowers were removed from the plant, and the volume was measured with a graduated 50 µL syringe.

### Genotyping

Plant leaves were sampled and dried in silica gel, and DNA was extracted with the Qiagen DNeasy Plant Mini Kit (Qiagen, Mississauga, Canada). 300 ng of DNA was used to genotype individuals using a Genotyping By Sequencing approach, following the protocol developed by Elshire et al. (2011). Library preparation was performed at Laval University (IBIS plateform, Quebec city, Canada) using the restriction enzymes *Pst*I and *Msp*I. We sequenced 177 F2s, duplicating ten individuals to assess genotyping repeatability: four F2s, both parents, the self-pollinated F1 and three other F1s, and two progenies of the self-pollinated parents. Individuals were multiplexed in pools of 96 samples, and sequenced on two lanes on an Illumina Hiseq 2500 at McGill University and Génome Québec Innovation Centre (Montreal, Canada). Stacks pipeline version 1.20 Beta was used to extract genotypes from raw reads (Catchen et al., 2011). Reads were first demultiplexed and trimmed to 82 basepairs with the function process-radtags. Then, unique stacks were generated with the function ustacks, constraining for a minimum read depth (−m) of 2 to create a stack, and a maximum inter-read Single Nucleotide Polymorphism (SNP) distance (−M) of 5. The catalog was created with both parents, and SNP calls were first performed with default parameters in sstacks. Then, the error correction module rxstacks was run to perform automated corrections using the bounded SNP model and a cutoff ln likelihood value of −10 to discard unlikely genotypes. The cstacks and sstacks were then repeated with the corrected data, and genotypes data were obtained with the function genotypes. After running genotypes with the—GEN output format and allowing automatic corrections with default parameters, a R script was run to translate those data in an A (parent *R. auriculatum* allele), B (parent *R. rupincola* allele), H (heterozygous) format needed for subsequent analyses. In this script, using the information available from the self-pollinated F1, markers that are aaxab in the parents, and for which the F1 is ab were typed in the F2 population, an option not available in the Stacks pipeline.

Because mutations in some TCP genes are known to be involved in the determination of flower symmetry and in the size and shape of corollas (Hileman & Cubas, 2009), the genes *RADIALIS* and *CYCLOIDEA* were included in the linkage map to test if they could be involved in the variation in flower morphology between the two species. Gene sequences acquired from GenBank (sequence AY363927.1 from *R. auriculatum* for *Gcyc* and sequence AY954971.1 from *Antirrhinum majus* for *RADIALIS*) were compared to the parents’ transcriptomes (E Gonzalez, 2013, unpublished data) using BLASTn (Camacho et al., 2009) and primers were designed using software Primer3 (Koressaar & Remm, 2007). Gene sequences were deposited in Genbank (accession numbers KP794058, KP794059, KP794060, and KP794061).

*CYCLOIDEA* was genotyped with the CAPS method (Konieczny & Ausubel, 1993). Around 1 ng of DNA was added to a master mix containing 0.375 U of DreamTaq (Termoscientific, Waltham, Massachusetts, USA), 1.5 µL of 10X DreamTaq Buffer, 0.6 µL of each 10 µM primer and 0.3 µL of 10 mM dNTPs in a total reaction volume of 15 µL. Primers used to amplify *CYCLOIDEA* were gcycf2 (AAGGAGCTGGTGCAGGCTAAGA) and gcycr2 (GGGAGATTGCAGTTCAAATCCCTTGA), amplification conditions were 2 min at 94 °C, followed by 40 cycles of 94 °C 15 s, 54 °C 15 s, 72 °C 30 s, and then a final extension step of 1 min at 72 °C. Circa one µg of PCR product was then digested with *Afl*II (New England Biolabs, Ipswich, Massachusetts, USA) in a 15 µL volume according to the company’s recommendations. The total volume of digestion products was visualized on agarose gel. *RADIALIS* was genotyped with KASPAR (LGC genomics, Teddington, UK), with protocol tuning done by LGC genomics. DNA amplification was done with 75 ng of DNA, 2.5 µL of KASP master mix, and 0.07 µL of KASP primer mix in a total volume of 5 µL. The specific primer for the first parental allele was labelled with a FAM fluorochrome while the second specific primer was labelled with a HEX fluorochrome. Amplification conditions were a first step of 94 °C for 15 min, followed by 10 cycles of 94 °C 20 s, 61 °C decreasing of 0.6 °C at each cycle 1 min, and then another 29 cycles of 94 °C 20 s 55 °C 1 min. Genotypes were visualized by fluorescence after the amplification procedure on viia7 system (Applied Biosystems, Foster city, California, USA) with the “genotyping” protocol.

### Linkage map construction

GBS markers were filtered to keep only those with less than 25% data missing, and no segregation distortion (*χ*^2^ *p*-value >0.05 after Bonferonni correction). A linkage map was built with Carthagene (De Givry et al., 2005). Linkage groups were detected with a maximum two points distance of 30 centimorgan (cM) measured with Haldane function and a minimum LOD of 3. Marker ordering in each linkage group was done with the function *lkhd*, which implements the Lin-Kerninghan heuristic research algorithm to resolve the travelling salesman problem, optimising the 2 points distances along the linkage group. Once the first map was obtained, manual corrections were made for double-recombinants occurring within 10 cM. Because SNP calls can be erroneous if read depth is small, double recombinants scored as either A or B (homozygous) were replaced into H (heterozygous) if read depth was less than 10 reads. If read depth was more than 10, homozygous double recombinants were replaced by missing data as proposed by Kakioka et al. (2013), because those genotypes have a great probability of being erroneously typed. H (heterozygous) double recombinants were not replaced if both alleles were effectively detected in the sequencing data, but were replaced into A or B if only one allele was detected in the data (this case occurred because of mistakenly corrected calls from automatic correction in stacks). Remaining markers were then filtered again for missing data and segregation distortion, and a new map was built. This was repeated until no double-recombinants within 10 cM were found in the linkage map. After these cleaning steps, genotypes of both candidate genes were included in the dataset, and a final linkage map was built.

### QTL detection

Before performing QTL detection, correlation between colour, nectar volume and shape traits was tested in the F2 population using pearson coefficient for quantitative traits correlation and *F*-tests for colour. Among the 177 individuals, 141 gave flowers and were kept for colour tests. One hundred and thirty individuals were kept for shape QTL detection after inappropriate data was removed (see Phenotypic measurements section). Nectar volume was transformed into a binary trait for QTL detection given its large intra-individual variation and non-normal distribution in the F2 population (difference between the maximum and minimum volume for each individual ranged from 4 to 64 µL with a mean difference value of 25 µL). Individuals with mean volume inferior to 15 µL were classified as “0” and those with a mean volume superior to 25 µL as “1,” leaving 67 individuals to detect QTLs for nectar volume. QTL detection was performed with R/qtl version 1.33-7 (Broman et al., 2003). Genotypes probabilities were calculated every 1 cM with the function *calc.genoprob*. QTLs were looked for with *scaneone* with the normal model and the Haley-Knott method for the quantitative traits whereas the binary model and the EM method were used for nectar volume and colour. LOD scores were compared to the LOD threshold value obtained with 10,000 permutations. Then, if a QTL was detected, it was added as an additive covariate and the procedure rerun to detect minor QTLs. For non-binary variables, percentage of variance explained by the QTLs and size effects were checked with *fitqtl*, adding in the model one QTL at a time. Given the limited number of individuals scored for nectar volume, a supplementary Spearman correlation test between nectar volume (codes 0/1) and genotypic data for each marker (codes 1/2/3) was performed to confirm the QTL results.

### Pleiotropy and epistasy detection

Pleiotropic QTLs were searched by considering the principal axes of the PCA performed on the hybrid population as proposed by Mangin, Thoquet & Grimsley (1998). The computation of the pleiotropic test statistics was limited to the first three principal axes, which explained most of the variance, as suggested by Weller, Wiggans & VanRaden (1996). Briefly, the test was obtained by computing the LOD scores for each principal component and summing the result of all the three principal components. To access the threshold value of the pleiotropic test statistics, 10,000 permutations were performed (Doerge & Churchil, 1996) with the three principal components being permutated all together in order to get a null distribution, while preserving the initial intra-individual relation between phenotypic traits. QTL detection was based on the 95th quantile. Confidence regions were estimated with a 2-LOD support, as suggested by Van Ooijen (1992). Epistasy among QTLs as well as among QTLs and other markers were tested using MCQTL (Jourjon et al., 2005).

Scripts and data used for morphometric analyses, map building and QTL detection are available as Data S1.

## Results

### Correlation between traits and morphological variation

**Figure 3:**
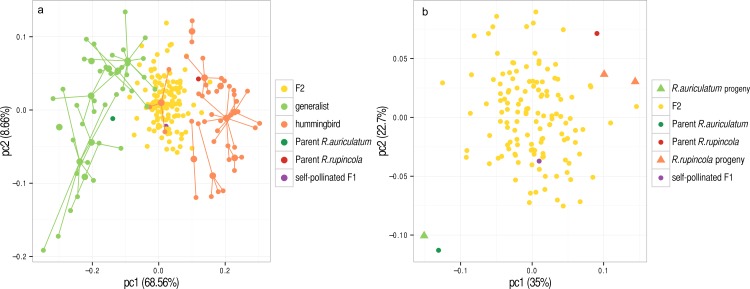
Principal component analyses of shape. (A) PCA performed on wild specimens from species with different pollination syndromes (method i—*Pollination syndrome differences*); Large and small dots represent species mean shapes and individual shapes, respectively, and individuals that belong to a given species are linked to it with a line. (B) PCA performed on the hybrid population (method iii–*Morphological variation in the hybrid population*) where triangles represent self-pollinated parents’ progeny. Numbers between brackets are percentage of shape variance represented by each axis.

For the PCA performed on the 18 species with divergent pollination syndromes (approach i), the first principal component (PC1: 68.55%) discriminated hummingbird specialist species from generalists (Fig. 3A). Both parents were positioned within their respective pollination syndrome group while the self-pollinated F1 and the F2 population were intermediate between both syndromes for the first principal component. As only the first principal component separated the two syndromes, only this component was used for QTL detection. The first three principal components of the PCA performed on the hybrid population (approach iii) explained the majority of morphological variability found in the hybrid population (PC1: 35%, PC2: 22.7%, PC3: 14.2%, total = 71.9%; Fig. 3B). For this PCA, parents were at the extremities of the distribution, while the self-pollinated F1 and the F2s were intermediate between parents. Interestingly, the F2 individuals were closer to *R. rupincola* than *R. auriculatum* (Fig. 3B).

**Figure 4:**
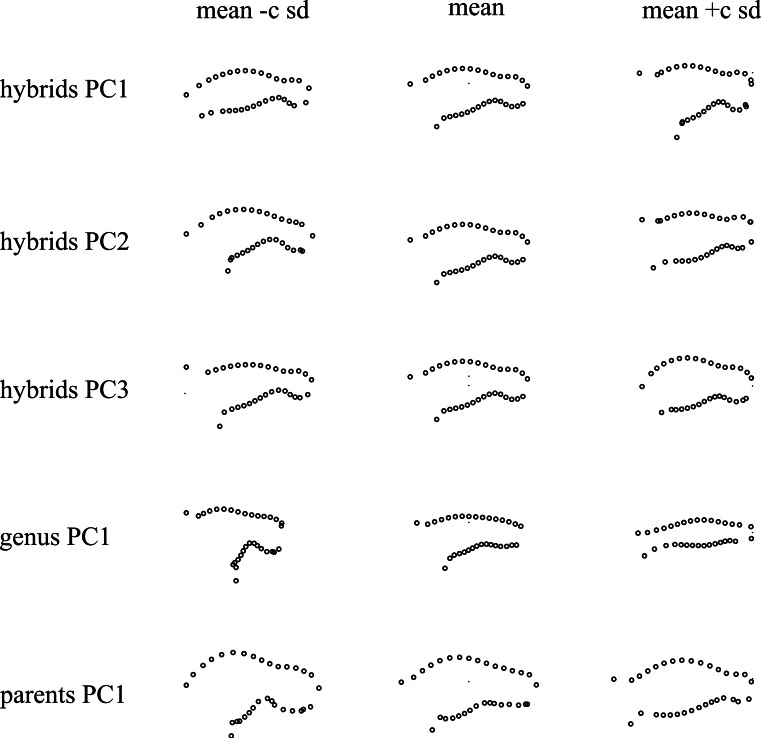
Shape variation associated with each principal component. Each point represent a landmark (or semi-landmark) position on the profile of the corolla, as shown in Fig. 1B. Sd, standard deviation, *c* = 1 for hybrid population PCA, 0.5 for between syndrome PCA and 0.2 for between parents PCA.

**Figure 5:**
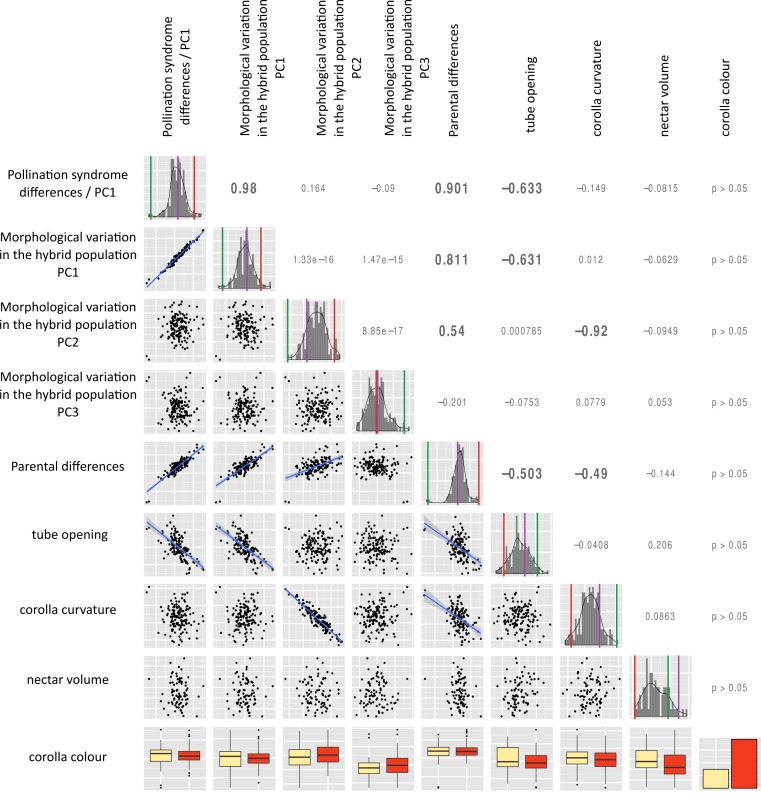
Distribution and correlation among traits in the hybrid population. Diagonal: traits distribution, the vertical lines correspond to the values of parent *R. rupincola* (red), parent *R. auriculatum* (green) and the self-pollinated F1 (purple). Lower left triangle, correlation among traits, if covariation is significant after Sidak correction, the regression line was plotted; Upper right triangle, regression coefficient, in bold if correlation is significant.

The correlation between the morphological principal components, two univariate traits (constriction size, the corolla curvature) and two binary traits (corolla colour, nectar volume) were measured. Traits corresponding to different pollination syndrome components (shape, colour, nectar) were not correlated among individuals of the F2 population (Fig. 5). However, the first principal component of each PCA (performed on the genus, both parents or the hybrid population) were correlated with each other with high correlation coefficient (first PC on the genus—first PC on the hybrid population: *r* = 0.98; first PC on the genus—PC on the parents: *r* = 0.901; first PC on the hybrid population—PC on the parents: *r* = 0.811, Fig. 5). Principal components of PCA were also sometimes correlated with univariate shape measures (second PC on the hybrid population-corolla curvature: *r* = − 0.92; constriction size-first PC on the genus: *r* = − 0.633, Fig. 5), and this correlation is also visible on Fig. 4 as flowers at the extreme of PC1 harbour different opening size and flowers at the low extreme of hybrids PC2 are more incurved than flowers at the high extreme.

### Molecular data and linkage map

**Figure 6:**
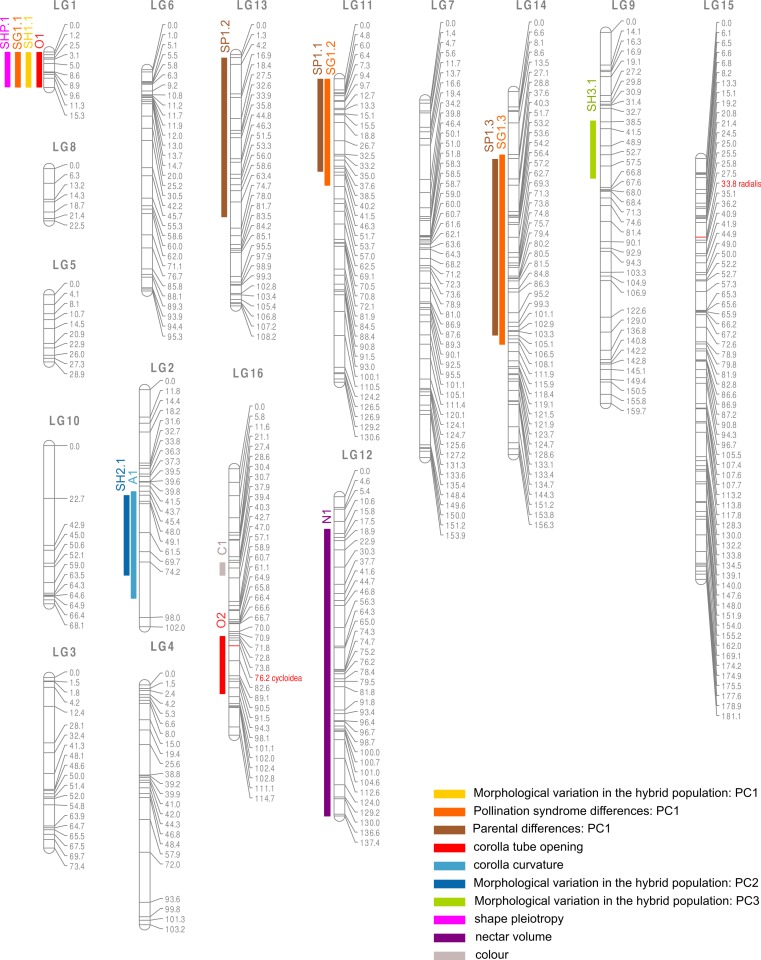
Linkage map and position of QTLs. QTLs positions are marked with 2-LOD confidence region, numbers right to the linkage groups represent markers position in cM. Unique names for QTLs correspond to the names given in Table 2.

Starting from ca. 422 millions raw reads, the stacks pipeline initially gave 2,257 markers. After removing markers with more than 25% missing data and with segregation distortion, 845 markers remained to construct a genetic map. Then, with a third step of iterative map building, following correction for double recombinants and filtering for missing data, we finally obtained 557 clean GBS makers plus the two candidate genes. With a maximum distance of 30 cM between consecutive markers and a minimum LOD score of 3, 16 linkage groups were identified. Groups remained stable even if the LOD threshold was changed from 1 to 10, which is suggestive of relatively good stability of our linkage groups. The linkage map represents a total length of 1650.6 cM with an average distance between adjacent markers of 3.39 cM and relatively heterogeneous linkage group size (Table 1 and Fig. 6). Recombination fractions and 2-points LOD scores can be visualised on Fig. S2.

**Table 1:** Information about linkage groups.

Linkage group	Number of markers	Size (cM)	Average distance between markers (cM)
LG1	12	15.3	1.7
LG2	23	102	4.86
LG3	22	73.4	3.86
LG4	26	103.2	4.49
LG5	11	28.9	3.21
LG6	36	95.3	3.07
LG7	59	153.9	3.02
LG8	7	22.5	3.75
LG9	46	159.7	4.2
LG10	13	68.1	5.68
LG11	48	130.6	3.19
LG12	40	137.4	3.71
LG13	34	108.2	3.49
LG14	57	156.3	3.13
LG15	81	181.1	2.62
LG16	44	114.7	2.94
Total	559	1650.6	3.39

### QTL analysis

#### QTLs for simple traits

The ratio of yellow to orange flowered individuals in the F2s was of 42:99, which is not significantly different from a 1:3 ration expected for a dominant Mendelian marker (*χ*^2^ test: *χ*^2^ = 1.7234; d.f = 1; *p*-value = 0.1893). A single QTL, on linkage group LG16, was found to explain colour variation in the F2 population (Fig. 6).

One QTL explaining nectar volume differences was detected on LG12, with a very large confidence region (123.4 cM). These results were confirmed by correlation between the traits and markers as only two markers, both on LG12, were significantly correlated to nectar after a Bonferonni correction (position 46.1, *p*-value = 2.78E–06; position 56.3, *p*-value = 4.19E–05). As for colour, the amount of variance explained by this QTL couldn’t be measured because the data were transformed (binary model).

Shape was analysed with geometric morphometrics and with univariate measures. For the shape variation between pollination syndromes (approach i) three distinct QTLs on LG1, LG11, and LG14 were detected and explained respectively 12.8%, 13.6% and 8.8% of variance (Fig. 6 and Table 2). For the shape variation between parents (approach ii) also three QTLs were detected on LG13, LG11 and LG14 explaining 6.7%, 10.2% and 12.8% of the variance (Fig. 6 and Table 2). When measuring morphological variation in the F2 hybrids (approach iii), one QTL was identified as controlling the first component on LG1 and explained 15.1% of the variance, another QTL on LG2 explained 14% of the variance for the second component, and a third QTL on LG9 explained the 14.9% of variance for the third component (Fig. 6 and Table 2). Corolla tube opening variation was explained by 2 QTLs on LG1 and LG16, explaining 12.5% and 12.4% of the variance, respectively. Corolla curvature was underlain by one QTL on LG2 explaining 12.8% of the variance.

**Table 2:** Position and effects of QTLs. Positions are given in centimorgan (cM) from the beginning of the linkage group; confidence regions are calculated with 2 LOD score decrease. QTL names are the same as in Fig. 6. The relative homozygous effect is the additive effect divided by the mean phenotypic difference between both parents.

Trait		QTL name	Linkage group	Position	Confidance region	Variance % explained	Additive effect	Relative homozygous effect	*t* value
Colour		C1	LG16	43	40.3–46	–	–	–	–
Nectar volume		N1	LG12	41.6	14–137.4	–	–	–	–
PCA on hybrid population	pc1	SH1.1	LG1	11.3	0–15.3	15.13	0.023	0.10	4.487
	pc2	SH2.1	LG2	57	45.4–80	14.06	0.020	0.11	4.120
	pc3	SH3.1	LG9	19	38.63	14.90	−0.017	0.19	−4.705
	Pleiotropy	SHP.1	LG1	0	0–15	–	–	–	–
PCA on parents	pc1	SP1.1	LG11	28	0–40	10.26	0.017	0.05	4.209
		SP1.2	LG13	18	1.3–70	6.68	0.013	0.04	3.493
		SP1.3	LG14	91	29–105	12.88	0.015	0.05	4.347
PCA on genus	pc1	SG1.1	LG1	11.3	0–15.3	12.76	0.020	0.08	4.698
		SG1.2	LG11	29	0–46	13.60	0.023	0.09	4.938
		SG1.3	LG14	86.3	27.1–109	8.85	0.015	0.06	3.625
Corolla curvature		A1	LG2	54	43.7–90	12.84	−5.987	0.10	−3.684
Corolla tube opening		O1	LG1	15.3	0–15.3	12.48	−0.061	0.14	−4.591
	O2	LG16	85	72–97	12.36	−0.066	0.15	−4.119

Interestingly, the same QTLs were detected irrespective of the way morphology was quantified (Fig. 6), that is, co-localizing QTLs were detected for co-varying traits. For instance, the QTL on LG1 was detected with the different methods used to measure shape. Specifically, it was detected using the principal component that distinguished generalists and specialists as well as using corolla tube constriction. Considering all shape analyses together, a total of seven different QTLs were detected, which explained a small to moderate part of morphological variance (Table 2).

We found that one candidate gene for floral shape, *CYCLOIDEA* (at position 76.2 cM on LG16), co-localized with a QTL confidence region for corolla constriction, although the position of the gene does not correspond to the maximum LOD value (which corresponds to position 85 cM, Fig. 6 and Table 2). *RADIALIS* did not co-localize with any QTL.

#### Pleiotropic and epistatic QTLs

When analyzing QTLs acting pleiotropically on the first three shape components obtained from the PCA on the hybrid population, one QTL was detected on LG1, co-localizing with QTLs for simple traits. Epistasy analysis was conducted with MCQTL and no epistatic interaction was detected among QTLs and neither among QTLs and other markers.

## Discussion

### Detection of moderate QTLs involved in pollination syndrome transition

Our linkage map construction was able to recover 16 linkage groups. This is two more than the haploid chromosome number (*n* = 14) for *Rhytidophyllum* (Skog, 1976). However, while karyotype information exists for *R. auriculatum* (Skog, 1976), none exist specifically for *Rhytidophyllum rupincola*. Yet, an *n* = 14 for *R. rupincola* appears likely because all *Rhytidophyllum* species studied so far are *n* = 14. In addition, differences in chromosome number between the parents seem unlikely given the viability of second generation hybrids. Finding more linkage groups than chromosomes might result from low genome coverage, however, we do not favour this hypothesis as the average distance between consecutive markers is of 3.39 cM. The parents of the cross are from distinct species, and chromosomal rearrangements could have occurred between them. These could create difficulties in assigning some chromosomal segments to the rest of the chromosome; the smallest linkage groups could thus correspond to rearranged chromosomal regions between both species.

#### Colour differences QTL

We detected one QTL explaining colour transition between *R. auriculatum* and *R. rupincola*. The results presented here are consistent with previous studies on pollination syndrome transitions that investigated the genetic basis of colour variation. Wessinger, Hileman & Rausher (2014) found one QTL for colour, corresponding to a gene involved in anthocyanin biosynthesis pathway. Similarly, the well-known case of colour transition between *Mimulus lewisii* and *M. cardinalis* showed that a single mutation at the *YUP* locus can both affect flower colour and pollinators behaviour (Bradshaw & Schemske, 2003). However, an important variation of colour patterns among orange flowers was observed in the hybrid population, both in terms of intensity and localisation of pigments (Fig. 1). This suggests that other genes could be involved in the intensity and distribution pattern of pigments, probably through differential gene expression over the corolla. Other studies on the genetic basis of colour transitions suggests that colour transitions generally involves down-regulation of genes of pigment biosynthesis pathway, often via the action of transcription factors (Galliot, Stuurman & Kuhlemeier, 2006). Future work will then involve the study of the association between colour pattern and the expression of major genes in the anthocyanin biosynthesis pathway.

#### Nectar volume QTL

Categorizing individuals as “low producing” or “high producing,” and using a binary QTL detection model, permitted the detection of one QTL. We also tried to study sugar concentration in nectar (using a Hand Held Brix Refractometer 0–32°; Fisher Scientific International Inc., Hampton, New Hampshire, USA) but faced the same variability problems as for volume and did not succeed in detecting any QTL (data not shown). The confidence region of the QTL for nectar volume was very large. Other QTLs could likely be detected with a larger sample size and stricter growing conditions to decrease intra individual variation. Indeed, similar studies generally detected several QTLs explaining nectar volume variation. Bradshaw et al. (1998) detected two QTLs for nectar volume explaining together 63.4% of total variance. Similarly, Stuurman et al. (2004) also detected two QTLs associated with nectar volume in *Petunia* pollination syndromes. In contrast, Wessinger, Hileman & Rausher (2014) detected only one QTL for nectar volume variation.

#### Multiple QTLs for corolla shape

Floral shape was measured in order to first understand the genetic basis of the component of corolla shape associated with pollination syndrome transition and second, to understand the genetic basis of the components of corolla shape that are representative of differences between both parents, but not necessarily important for pollination syndrome identity. For the shape component defined by pollination mode differences, three independent QTLs were detected. While only few QTLs were expected for this shape component, to our knowledge, no other studies have successfully identified QTLs for pollination syndrome with geometric morphometrics and PCA methods. However, we can compare our results with studies analysing shape differences in divergent environments in other organisms. For instance, Franchini et al. (2014) used the same method to study the relationship between body shape evolution and trophic ecology between two fish species. Their results are similar to ours in that they also detected relatively few QTL (4), each one explaining less than 8% of variance.

Regarding shape differences that are not necessarily associated with pollination syndromes (obtained with the PCAs on the parents and on the hybrid population), seven distinct QTLs were detected. Removing those that co-localized with QTLs found with shape differences associated with the pollination syndromes, four shape QTLs remained. Conceivably, shape may have initially evolved dramatically during the process of pollination syndrome transition, followed by gradual, small changes along the evolutionary tree (the two species studied are not sister species). Such a hypothesis could be tested with repeated QTL studies involving closely related species and phylogenetic comparative methods (Moyle & Payseur, 2009).

These results suggest that the genetic basis of shape evolution is more complex than those of colour and nectar volume. Other studies of floral morphology detected multiple QTLs with small to moderate effects explaining morphological changes linked to pollination syndrome evolution, supporting this idea of increased complexity. For example, Hodges et al. (2002) detected multiple QTLs for spur length and flower orientation differences between two *Aquilegia* species. Wessinger, Hileman & Rausher (2014) detected multiple QTLs between two *Penstemon* species associated with morphological differences (explaining between 7.3 and 24.3% of the shape variance). Galliot et al. (2006) detected six QTLs explaining several component of flower size in *Petunia* representing each 2.7 to 41.6% of the variance, as well as four QTLs for nectar volume (explaining 4.2 to 39.1% of the variance). The same was detected for five morphological traits in *Leptosiphon* (each one represented by two to seven QTLs explaining two to 28% of the variance) (Goodwillie, Ritland & Ritland, 2006).

A candidate gene approach is always interesting as it can provide clues as to which genes might be involved in the morphological variation observed in a system. *RADIALIS* and *CYCLOIDEA* are two genes thought to be involved in the determination of floral zygomorphy (Preston, Martinez & Hileman, 2011). More specifically, their localized expression during flower development determines petal size and shape (Feng et al., 2006; Luo et al., 1999; Wang et al., 2008). As such, they represented strong candidate genes in the present study for controlling floral shape, especially for the curvature of flowers. Neither were found to be clearly linked to the morphological differences between *R. auriculatum* and *R. rupincola*. Indeed, although *CYCLOIDEA* is situated within the confidence region of one QTL explaining corolla tube opening, it had a LOD score that did not pass the rejection threshold (Fig. 6 and Fig. S2) and is therefore unlikely to be highly involved in trait variation. This suggests that these candidate genes are not directly responsible for corolla shape variation in our system, at least not for the major shape differences between the pollination syndromes or in the hybrid population. However, this does not mean that they are not involved at all as critical changes could involve the regulation of their expression. If these candidate genes are trans-regulated, then the QTL would not be expected to localize with them. Clearly, further studies will be needed to better understand the genetic basis of flower shape variation in this system.

### Pollination syndrome evolution in the genus is summarized by morphological transition between *R. auriculatum* and *R. rupincola*

Our results showed that generalist and hummingbird specialist species can be differentiated with only one shape component in *Rhytidophyllum* and *Gesneria*, which concurs with a broader study of the group (F Lambert, 2015, unpublished data). This shape component correlates with corolla tube opening in our hybrid population and discriminates the columnar shape of hummingbird pollinated species from the cup shape of generalists (Martén-Rodríguez, Almarales-Castro & Fenster, 2009). The strong correlation between the shape components obtained with the PCA at the genus level, among the parents and on the F2 hybrid population suggest that similar drivers may lie behind flower shape variation. This implies that morphological transition between *R. auriculatum* and *R. rupincola* is representative of the major morphological disparity between pollination syndromes at the genus level.

To compare the genetic basis of simple traits and global shape, both univariate traits and multivariate traits were measured. Our results showed that univariate morphological traits were strongly correlated with geometric morphometric shape components, suggesting that the information contained in simple traits is generally contained in geometric morphometrics data. Moreover, geometric morphometric approaches allowed the detection of more QTLs, demonstrating that they contain more information than simple traits. However, one QTL obtained with an univariate trait (QTL on LG16 for corolla tube opening) was not detected with geometric morphometric approaches. This could suggests that due to their complexity, geometric morphometric traits may not catch exactly the same variation as univariate traits, but in the present study these results could also be caused by lack of statistical power due to the small segregating population size. Our results are similar to those of Franchini et al. (2014) who also detected similar QTLs for geometric morphometric and simple traits measured with inter-landmark distances. Their study, however, also showed that additional QTLs were identified for univariate characteristics obtained independently from landmark data. Altogether, although these observations strongly favour the use of geometric morphometric in QTL studies, they reveal that it could also be beneficial to include univariate traits when analysing genetic architecture of shape evolution, particularly when using small population sizes.

### The role of selection in pollination syndrome transition

For most traits measured, only one major QTL was detected. However, for corolla tube opening, inter-parents PC and inter-syndromes PC, two, three, and three QTLs were detected, respectively. All the effects of these QTLs had the same direction. While such a result cannot be validated statistically because of the small number of QTLs, it suggests that those traits evolved under directional selection rather than by drift.

The existence of relationships between traits could impact the rate of adaptation. While some authors argue that genetic correlation between traits could slow down adaptation, other showed that it can facilitate it (reviewed in Hendry, 2013). Several studies found more or less important correlations between some traits involved in pollination syndrome transitions. Wessinger, Hileman & Rausher (2014) found no correlation between shape components, flower colour or nectar volume, although they found weak but significant correlations between nectar concentration and some morphological traits. Galliot et al. (2006), in their segregating *Petunia* population, detected a correlation between nectar volume and floral tube width. In a study of monkeyflowers, Bradshaw et al. (1998) detected epistatic interactions between the locus *YUP* involved in flower colour via carotenoid concentration and two other putative QTLs. Hermann et al. (2013), who studied QTLs of pollination syndromes in *Petunia*, found that QTLs involved in flower scent, colour and morphology were tightly clustered in one genomic region.

In our study, flower shape was found to be totally independent from colour and nectar characteristics. Detected QTLs for nectar, colour and shape are localized on different linkage groups or on different regions of the same group, suggesting these traits are genetically independent. However, it is not possible to completely rule out genetic correlations between traits for two reasons. Firstly, some correlations among floral characters might exist in our study system, but we didn’t measure components that are linked with each other (such as nectar concentration, pigments intensity and patterning on the corolla, style and stamen length, etc.). Secondly, correlations could involve minor QTLs that were not detected because of our small F2 population. It is also possible that strong correlations do not exist in our system. In such a case, we could consider that neither genetic constraints nor canalization played an important role in the pollination syndrome transition between *R. rupincola* and *R. auriculatum*. This would tend to show that selection pressure exerted by pollinators—that is, extrinsic factors—played a greater role in pollination syndrome evolution than intrinsic factors. Indeed, selection could have been exerted independently on each trait, and no developmental mechanism seems to have forced concerted evolution of pollination syndrome traits.

However, we still wonder if the same sequence of trait evolution could have taken place with replicated evolutions in the whole group? This question could be answered with replicated QTL studies on independent transitions and with the help of phylogenetic comparative methods. Accordingly, we agree with Moyle & Payseur (2009) that propose a combination of comparative methods with QTL analysis to better understand evolutionary patterns of reproductive isolation or evolution at a larger scale.

### Conclusion

The present study enabled the detection of major QTLs underlying the three major traits composing divergent pollination syndromes between two *Rhytidophyllum* species. Even if several minors QTLs potentially remain undetected, few major and independent regions for pollination syndrome transition were identified. The hypothesis raised by our study is that directional selection pressure exerted by different pollinators, rather than developmental constraints, was strong enough to make the different traits converge on a pollination syndrome.

## Supplemental Information

10.7717/peerj.1028/supp-1 Figure S1 Recombination fraction and LOD scores for each pair of markers Markers are in the same order as in the linkage map of Fig. 6; LOD scores are in the upper triangle and recombination fraction in the lower one. Colours represent a gradient from low LOD score and great recombination fraction (blue) to large LOD scores and small recombination fraction (red).

10.7717/peerj.1028/supp-2 Figure S2 Profiles of LOD scores for linkage groups with QTLs detected Abscises are marker position along the linkage group. Red lines are detection threshold corresponding to a type 1 error of 5%, and green rectangles correspond to the 2-LOD confidence region.

10.7717/peerj.1028/supp-3 Table S1 Information about species used for the PCA performed on the genus H, hummingbird specialist; G, generalist; (+1) correspond to parental individuals of the hybrid population but were not used to do the PCA.

10.7717/peerj.1028/supp-4 Data S1 Scripts and data used for the analyses

## Additional Information and Declarations

### DNA Deposition

The following information was supplied regarding the deposition of DNA sequences: Gene sequences were deposited in Genbank (accession numbers KP794058, KP794059, KP794060, and KP794061.

## References

[ref-1] Adams DC, Otárola-Castillo E (2013). Geomorph: an R package for the collection and analysis of geometric morphometric shape data. Methods in Ecology and Evolution.

[ref-2] Ashman T-L, Majetic CJ (2006). Genetic constraints on floral evolution: a review and evaluation of patterns. Heredity.

[ref-3] Bradshaw HD, Otto KG, Frewen BE, McKay JK, Schemske DW (1998). Quantitative trait loci affecting differences in floral morphology between two species of monkeyflower (*Mimulus*). Genetics.

[ref-4] Bradshaw HD, Schemske DW (2003). Allele substitution at a flower colour locus produces a pollinator shift in monkeyflowers. Nature.

[ref-5] Broman KW, Wu H, Sen S, Churchill GA (2003). R/qtl: QTL mapping in experimental crosses. Bioinformatics.

[ref-6] Camacho C, Coulouris G, Avagyan V, Ma N, Papadopoulos J, Bealer K, Madden TL (2009). BLAST+: architecture and applications. BMC Bioinformatics.

[ref-7] Catchen JM, Amores A, Hohenlohe P, Cresko W, Postlethwait JH (2011). Stacks: building and genotyping Loci de novo from short-read sequences. G3.

[ref-8] De Givry S, Bouchez M, Chabrier P, Milan D, Schiex T (2005). CARTHAGENE: multipopulation integrated genetic and radiation hybrid mapping. Bioinformatics.

[ref-9] Doerge RW, Churchil GA (1996). Permuation tests for multiple loci affecting a quantitative character. Genetics.

[ref-10] Dray S, Dufour AB (2007). The ade4 package: implementing the duality diagram for ecologists. Journal of Statistical Software.

[ref-11] Dryden IL (2014). Shapes: statistical shape analysis.

[ref-12] Elshire RJ, Glaubitz JC, Sun Q, Poland JA, Kawamoto K, Buckler ES, Mitchell SE (2011). A robust, simple genotyping-by-sequencing (GBS) approach for high diversity species. PLoS ONE.

[ref-13] Feng X, Zhao Z, Tian Z, Xu S, Luo Y, Cai Z, Wang Y, Yang J, Wang Z, Weng L, Chen J, Zheng L, Guo X, Luo J, Sato S, Tabata S, Ma W, Cao X, Hu X, Sun C, Luo D (2006). Control of petal shape and floral zygomorphy in Lotus japonicus. Proceedings of the National Academy of Sciences of the United States of America.

[ref-14] Fenster CB, Armbruster WS, Wilson P, Dudash MR, Thomson JD (2004). Pollination syndromes and floral specialization. Annual Review of Ecology, Evolution, and Systematics.

[ref-15] Franchini P, Fruciano C, Spreitzer ML, Jones JC, Elmer KR, Henning F, Meyer A (2014). Genomic architecture of ecologically divergent body shape in a pair of sympatric crater lake cichlid fishes. Molecular Ecology.

[ref-16] Friedman J, Barrett SCH (2009). Wind of change: new insights on the ecology and evolution of pollination and mating in wind-pollinated plants. Annals of Botany.

[ref-17] Galliot C, Hoballah ME, Kuhlemeier C, Stuurman J (2006). Genetics of flower size and nectar volume in Petunia pollination syndromes. Planta.

[ref-18] Galliot C, Stuurman J, Kuhlemeier C (2006). The genetic dissection of floral pollination syndromes. Current Opinion in Plant Biology.

[ref-19] Goodwillie C, Ritland C, Ritland K (2006). The genetic basis of floral traits associated with mating system evolution in *Leptosiphon* (Polemoniaceae): an analysis of quantitative trait loci. Evolution; International Journal of Organic Evolution.

[ref-20] Hendry AP (2013). Key questions in the genetics and genomics of eco-evolutionary dynamics. Heredity.

[ref-21] Hermann K, Klahre U, Moser M, Sheehan H, Therese M, Kuhlemeier C (2013). Tight genetic linkage of prezygotic barrier loci creates a multifunctional speciation Island in *Petunia*. Current Biology.

[ref-22] Hermann K, Kuhlemeier C (2011). The genetic architecture of natural variation in flower morphology. Current Opinion in Plant Biology.

[ref-23] Hileman LC, Cubas P (2009). An expanded evolutionary role for flower symmetry genes. Journal of Biology.

[ref-24] Hodges SA, Whittall JB, Fulton M, Yang JY (2002). Genetics of floral traits influencing reproductive isolation between *Aquilegia formosa* and *Aquilegia pubescens*. The American Naturalist.

[ref-25] Jourjon MF, Jasson S, Marcel J, Ngom B, Mangin B (2005). MCQTL: multi-allelic QTL mapping in multi-cross design. Bioinformatics.

[ref-26] Kakioka R, Kokita T, Kumada H, Watanabe K, Okuda N (2013). A RAD-based linkage map and comparative genomics in the gudgeons (genus *Gnathopogon*, Cyprinidae). BMC Genomics.

[ref-27] Klingenberg CP (2010). Evolution and development of shape: integrating quantitative approaches. Nature Reviews Genetics.

[ref-28] Klingenberg CP, Leamy LJ, Routman EJ, Cheverud JM (2001). Genetic architecture of mandible shape in mice: effects of quantitative trait loci analyzed by geometric morphometrics. Genetics.

[ref-29] Konieczny A, Ausubel FM (1993). A procedure for mapping *Arabidopsis* mutations using co-dominant ecotype-specific PCR-based markers. The Plant Journal.

[ref-30] Koressaar T, Remm M (2007). Enhancements and modifications of primer design program Primer3. Bioinformatics.

[ref-31] Langlade NB, Feng X, Dransfield T, Copsey L, Hanna AI, Thébaud C, Bangham A, Hudson A, Coen E (2005). Evolution through genetically controlled allometry space. Proceedings of the National Academy of Sciences of the United States of America.

[ref-32] Liu J, Shikano T, Leinonen T, Cano JM, Li M-H, Merilä J (2014). Identification of major and minor QTL for ecologically important morphological traits in three-spined sticklebacks (*Gasterosteus aculeatus*). G3.

[ref-33] Luo D, Carpenter R, Copsey L, Vincent C, Clark J, Coen E (1999). Control of organ asymmetry in flowers of antirrhinum. Cell.

[ref-34] Mangin B, Thoquet P, Grimsley N (1998). Pleiotropic QTL analysis. Biometrics.

[ref-35] Martén-Rodríguez S, Almarales-Castro A, Fenster CB (2009). Evaluation of pollination syndromes in Antillean Gesneriaceae: evidence for bat, hummingbird and generalized flowers. Journal of Ecology.

[ref-36] Martén-Rodríguez S, Fenster CB (2008). Pollination ecology and breeding systems of five Gesneria species from Puerto Rico. Annals of Botany.

[ref-37] Martén-Rodríguez S, Fenster CB, Agnarsson I, Skog LE, Zimmer EA (2010). Evolutionary breakdown of pollination specialization in a Caribbean plant radiation. The New Phytologist.

[ref-38] Moyle LC, Payseur BA (2009). Reproductive isolation grows on trees. Trends in Ecology & Evolution.

[ref-39] Nakazato T, Rieseberg LH, Wood TE (2013). The genetic basis of speciation in the Giliopsis lineage of *Ipomopsis* (Polemoniaceae). Heredity.

[ref-40] Perret M, Chautems A, Spichiger R, Barraclough TG, Savolainen V (2007). The geographical pattern of speciation and floral diversification in the neotropics: the tribe Sinningieae (Gesneriaceae) as a case study. Evolution.

[ref-41] Preston JC, Martinez CC, Hileman LC (2011). Gradual disintegration of the floral symmetry gene network is implicated in the evolution of a wind-pollination syndrome. Proceedings of the National Academy of Sciences of the United States of America.

[ref-42] Quattrocchio F, Wing J, Van Der Woude K, Souer E, De Vetten N, Mol J, Koes R (1998). Molecular analysis of the anthocyanin2 gene of *Petunia* and its role in the evolution of flower color. The Plant Cell.

[ref-43] R Core Team (2014). R: a language and environment for statistical computing.

[ref-44] Roalson EH, Skog LE, Zimmer EA (2008). Untangling Gloxinieae (Gesneriaceae). II. Reconstructing biogeographic patterns and estimating divergence times among new world continental and Island lineages. Systematic Botany.

[ref-45] Rogers SM, Tamkee P, Summers B, Balabahadra S, Marks M, Kingsley DM, Schluter D (2012). Genetic signature of adaptive peak shift in threespine stickleback. Evolution; International Journal of Organic Evolution.

[ref-46] Rosas-Guerrero V, Aguilar R, Martén-Rodríguez S, Ashworth L, Lopezaraiza-Mikel M, Bastida JM, Quesada M (2014). A quantitative review of pollination syndromes: do floral traits predict effective pollinators?. Ecology Letters.

[ref-47] Skog LE (1976). A study of the tribe gesnerieae, with a revision of gesneria (Gesneriaceae: Gesnerioideae). Smithonian Contributions to Botany.

[ref-48] Stebbins GL (1970). Adaptive radiation of reproductive characteristics in angiosperms, I: pollination mechanisms. Annual Review of Ecology and Systematics.

[ref-49] Stuurman J, Hoballah ME, Broger L, Moore J, Basten C, Kuhlemeier C (2004). Dissection of floral pollination syndromes in *Petunia*. Genetics.

[ref-50] Tripp EA, Manos PS (2008). Is floral specialization an evolutionary dead-end? Pollination system transitions in *Ruellia* (Acanthaceae). Evolution; International Journal of Organic Evolution.

[ref-51] Van der Niet T, Johnson SD (2012). Phylogenetic evidence for pollinator-driven diversification of angiosperms. Trends in Ecology & Evolution.

[ref-52] Van Ooijen JW (1992). Accuracy of mapping quantitative trait loci in autoganous species. Theoretical and Applied Genetics.

[ref-53] Wang Z, Luo Y, Li X, Wang L, Xu S, Yang J, Weng L, Sato S, Tabata S, Ambrose M, Rameau C, Feng X, Hu X, Luo D (2008). Genetic control of floral zygomorphy in pea (*Pisum sativum* L.). PNAS.

[ref-54] Weller JI, Wiggans GR, VanRaden PM (1996). Application of a canonical transformation to detection of quantitative trait loci with the aid of genetic markers in multitrait experiment. Theoretical and Applied Genetics.

[ref-55] Wessinger CA, Hileman LC, Rausher MD (2014). Identification of major quantitative trait loci underlying floral pollination syndrome divergence in *Penstemon*. Philosophical Transactions of the Royal Society of London B: Biological Sciences.

[ref-56] Wilson P, Wolfe AD, Armbruster WS, Thomson JD (2007). Constrained lability in floral evolution: counting convergent origins of hummingbird pollination in *Penstemon* and *Keckiella*. The New Phytologist.

[ref-57] Yuan Y-W, Sagawa JM, Young RC, Christensen BJ, Bradshaw HD (2013). Genetic dissection of a major anthocyanin QTL contributing to pollinator-mediated reproductive isolation between sister species of *Mimulus*. Genetics.

