# Supplementary figures and images for: Genetic architecture of pollination syndrome transition between hummingbird-specialist and generalist species in the genus Rhytidophyllum (Gesneriaceae)
Source: PeerJ. 2015 Jun 18;3:e1028. doi: 10.7717/peerj.1028 (PMC4476130; doi:10.7717/peerj.1028)

## Pairwise recombination fractions and LOD scores

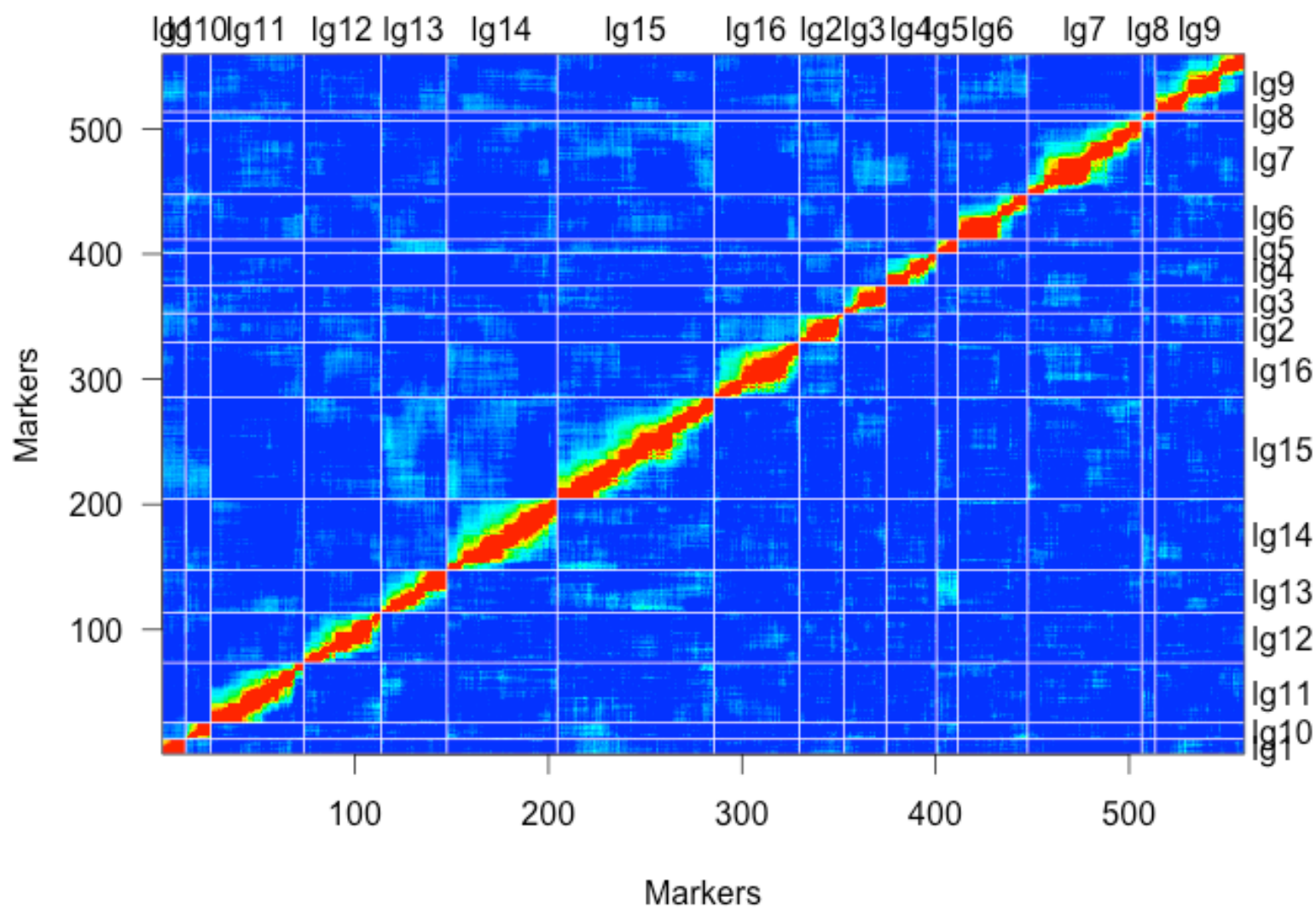

Supplement: Figure S1 — Markers are in the same order as in the linkage map of Fig. 6; LOD scores are in the upper triangle and recombination fraction in the lower one. Colours represent a gradient from low LOD score and great recombination fraction (blue) to large LOD scores and small recombination fraction (red). [file peerj-03-1028-s001.pdf]
