# Supplementary material for: Genetic architecture of pollination syndrome transition between hummingbird-specialist and generalist species in the genus Rhytidophyllum (Gesneriaceae)
Source: PeerJ. 2015 Jun 18;3:e1028. doi: 10.7717/peerj.1028 (PMC4476130; doi:10.7717/peerj.1028)

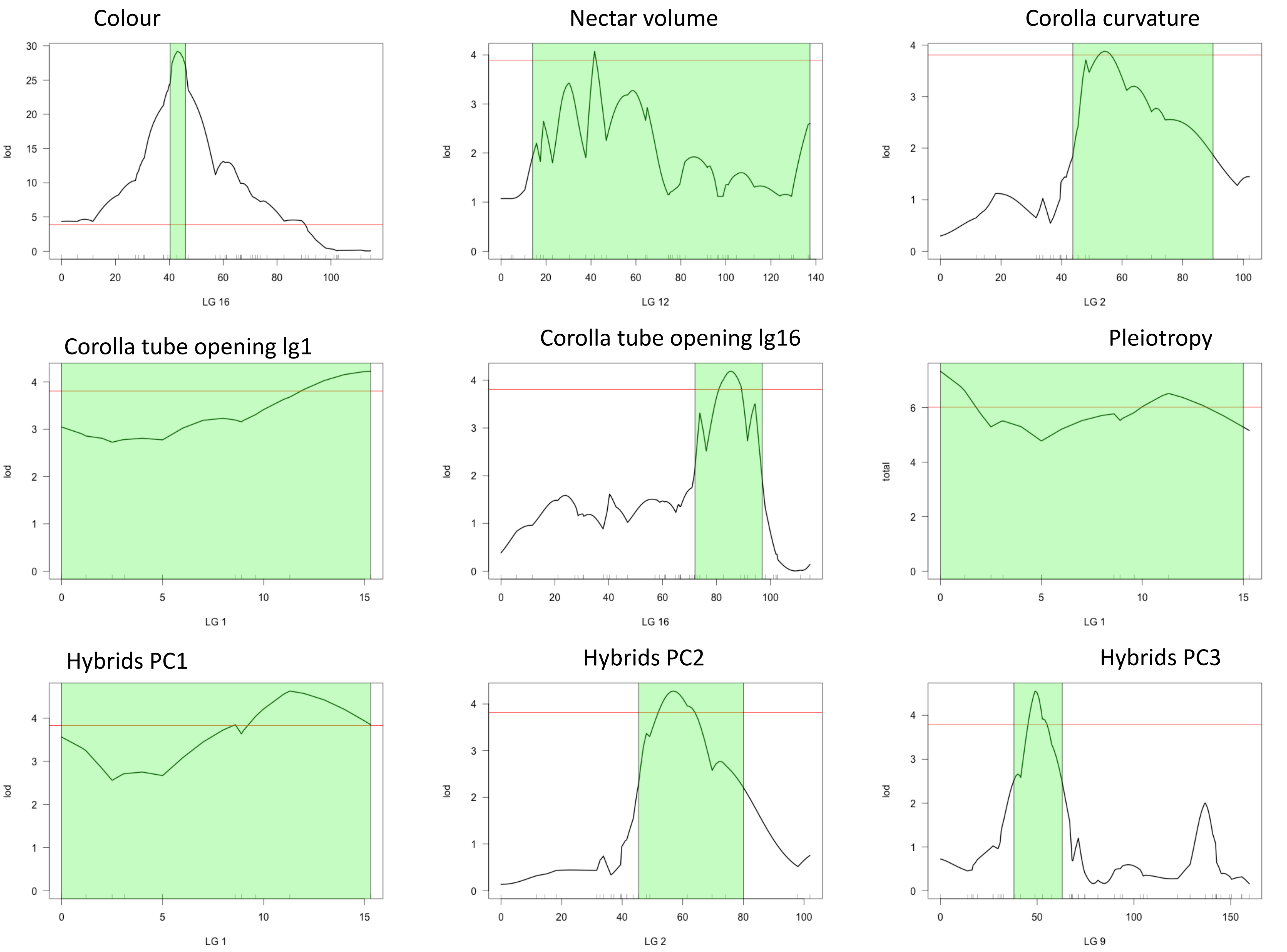

Colour
lod
LG 16
Nectar volume
lod
LG 12
Corolla curvature
lod
LG 2
Corolla tube opening lg1
lod
LG 1
Corolla tube opening lg16
lod
LG 16
Pleiotropy
total
LG 1
Hybrids PC1
lod
LG 1
Hybrids PC2
lod
LG 2
Hybrids PC3
lod
LG 9

Genus PC1

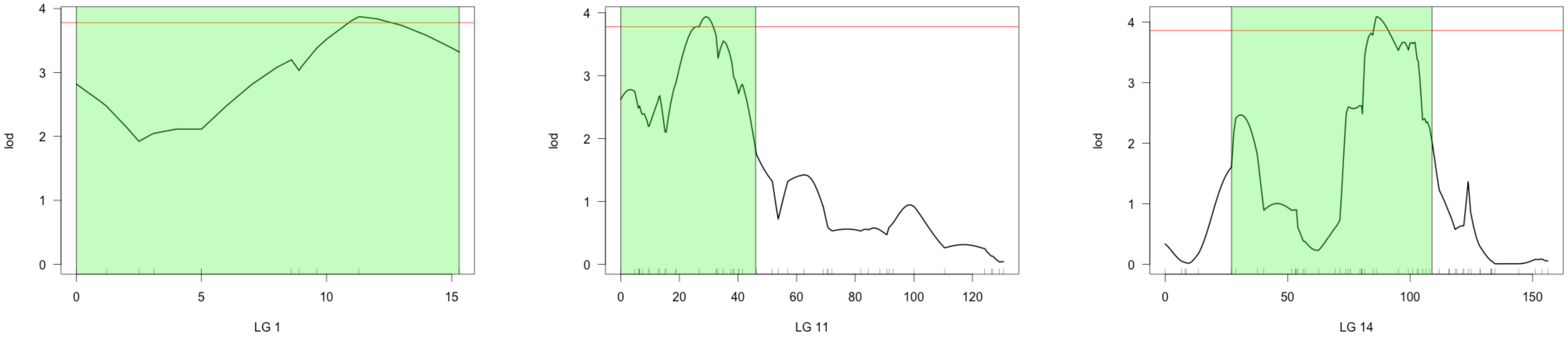

Parents PC1

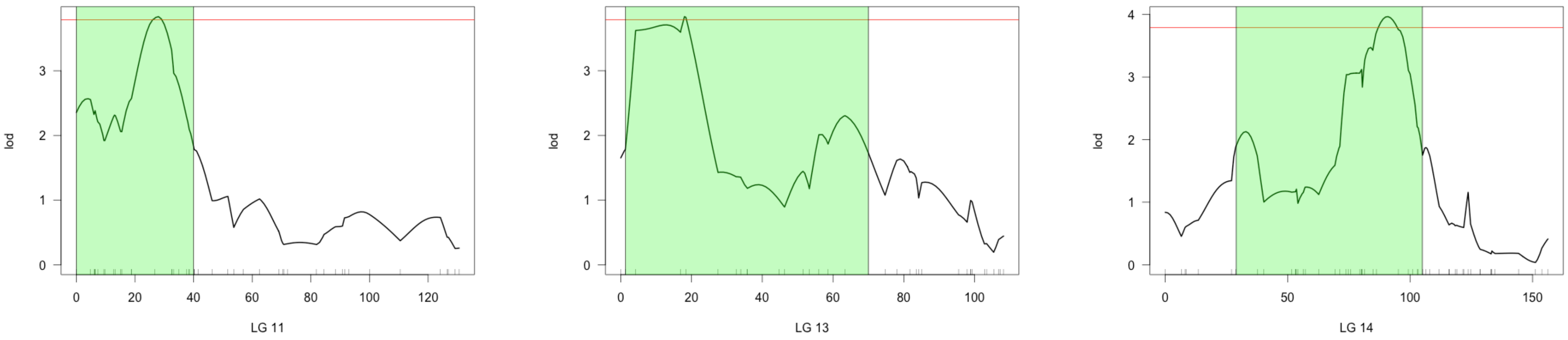

Supplement: Figure S2 — Abscises are marker position along the linkage group. Red lines are detection threshold corresponding to a type 1 error of 5%, and green rectangles correspond to the 2-LOD confidence region. [file peerj-03-1028-s002.pdf]
